# Differential Effect of Antioxidants Glutathione and Vitamin C on the Hepatic Injuries Induced by *Plasmodium berghei* ANKA Infection

**DOI:** 10.1155/2021/9694508

**Published:** 2021-09-04

**Authors:** Nayara Kauffmann, Luana K. R. L. da Penha, Danielle V. Braga, Brenda J. A. Ataíde, Nivia S. F. Mendes, Laiane P. de Sousa, Givago S. da Souza, Adelaide C. F. Passos, Evander J. O. Batista, Anderson M. Herculano, Karen R. H. M. Oliveira

**Affiliations:** ^1^Instituto de Ciências Biológicas, Laboratório de Neurofarmacologia Experimental, Universidade Federal do Pará, Belém, Pará, Brazil; ^2^Núcleo de Medicina Tropical, Laboratório de Neurologia Tropical, Universidade Federal do Pará, Belém, Pará, Brazil; ^3^Núcleo de Medicina Tropical, Laboratório de Protozoologia, Universidade Federal do Pará, Belém, Pará, Brazil

## Abstract

Malaria is a life-threatening disease caused by *Plasmodium* and represents one of the main public health problems in the world. Among alterations associated with the disease, we highlight the hepatic impairment resulting from the generation of oxidative stress. Studies demonstrate that liver injuries caused by *Plasmodium* infection are associated with unbalance of the antioxidant system in hepatocytes, although little is known about the role of antioxidant molecules such as glutathione and vitamin C in the evolution of the disease and in the liver injury. To evaluate disease complications, murine models emerge as a valuable tool due to their similarities between the infectious species for human and mice. Herein, the aim of this study is to evaluate the effect of antioxidants glutathione and vitamin C on the evolution of murine malaria and in the liver damage caused by *Plasmodium berghei* ANKA infection. Mice were inoculated with parasitized erythrocytes and treated with glutathione and vitamin C, separately, both at 8 mg/kg during 7 consecutive days. Our data showed that during *Plasmodium* infection, treatment with glutathione promoted significant decrease in the survival of infected mice, accelerating the disease severity. However, treatment with vitamin C promoted an improvement in the clinical outcomes and prolonged the survival curve of infected animals. We also showed that glutathione promoted increase in the parasitemia rate of *Plasmodium*-infected animals, although treatment with vitamin C has induced significant decrease in parasitemia rates. Furthermore, histological analysis and enzyme biochemical measurement showed that treatment with glutathione exacerbates liver damage while treatment with vitamin C mitigates the hepatic injury induced by the infection. In summary, the current study provided evidences that antioxidant molecules could differently modulate the outcome of malaria disease; while glutathione aggravated the disease outcome and liver injury, the treatment with vitamin C protects the liver from damage and the evolution of the condition.

## 1. Introduction

Malaria represents one of the major and oldest public health problems in developing countries [1, 2]. Despite a global fall in mortality rates since 2000, malaria remains a worldwide problem with 228 million cases and 405.000 deaths only in 2018 [3, 4]. In endemic areas, deaths by *Plasmodium* infection occur as a result of severe complications which include renal failure, acidosis, cerebral malaria, respiratory distress, and severe anaemia followed by liver impairment [5–7].

As widely described in literature, malaria disease is caused by infection with species of *Plasmodium* spp. This parasite presents a complex life cycle involving different stages of development in both mosquito and human hosts [8, 9]. Ookinetes and sporozoites are the life forms of *Plasmodium* found in a definitive mosquito host, and the sporozoites are responsible for infecting hepatocytes in the liver of a vertebrate host. Sporozoites reproduce asexually inside hepatocytes generating exoerythrocytic merozoites which migrate to bloodstream and infect blood erythrocytes [10, 11]. In the vertebrate host, the liver is considered a *Plasmodium* “depository” being an essential organ to the development of malaria disease. Malaria-associated liver injury is common in both adults and pediatric patients and is characterized by an increase in the serum levels of biochemical markers such as bilirubin and aminotransferases [12–14]. Furthermore, Kupffer cell hyperplasia, hemozoin deposition, and monocyte infiltration are also frequent histological alterations described in the malaria hepatopathy [15, 16].

Previous studies demonstrate that liver injuries caused by *Plasmodium* infection are associated with unbalance of the antioxidant system in hepatocytes [17]. However, it remains unclear if treatment with antioxidants is able to protect the liver of infected subjects. Glutathione (GSH) is the main antioxidant in intracellular environment, and the liver represents the most important provider of GSH to organs and tissues [18, 19]. It is also widely documented that vitamin C acts as an essential component for liver homeostasis [20]. Although few studies have demonstrated the beneficial effect of vitamin C on the malaria disease, there are no studies showing the effect of this compound on the histology and biochemistry of the infected liver. Similarly, it is not evidenced if treatment with GSH can exert a protective effect in the liver of *Plasmodium*-infected subjects. In this context, the current study was aimed at evaluating the effect of GSH and vitamin C treatments on the malaria outcome and in the liver injury of mice infected with *Plasmodium berghei* ANKA (PbA), which represent a well-established animal model of malaria [21, 22].

## 2. Materials and Methods

### 2.1. Animals

Male and female BALB/c mice (4-6 weeks old), weighing 20-25 g, were obtained from Animal Care Facilities of the Biological Sciences Institute, Federal University of Para. Animals were housed under specific pathogen-free conditions with fresh water and standard rodent food *ad libitum*. Mice were maintained in groups of 10 animals per cage at controlled room temperature (22-24°C) in a 12-hour light/dark cycle. Experiments were carried out in agreement with Institutional Animal Ethics Committee guidelines (Protocol Number 2229290317), and all efforts were made to minimize animal suffering.

### 2.2. *Plasmodium berghei* ANKA Infection and Antioxidant Treatment

Blood aliquots containing the *Plasmodium berghei* ANKA (PbA) strain were maintained in liquid nitrogen frozen stock solutions until experimental procedures. Mice were inoculated intraperitoneally (i.p.) with 10^6^ parasitized red blood cells (pRBCs), suspended in 200 *μ*l of phosphate-buffered saline (PBS; pH 7.4) as previously described by Ataide et al. [23]. Treatments with GSH (8 mg/kg/day diluted in 100 *μ*l PBS) or vitamin C (8 mg/kg/day diluted in 100 *μ*l PBS) were performed by i.p. injection one hour after infection with PbA. Control mice received the same volume of sterile phosphate-buffered saline solution. The treatment was performed daily, from day 1 to day 7 postinfection. Based on the treatment, mice (*n* = 28) were randomly assigned into four groups: uninfected control, PbA-infected mice, PbA-infected mice+8 mg/kg GSH, and PbA-infected mice+8 mg/kg vitamin C.

### 2.3. Survival Rate and Blood Parasitemia

Mice from the different groups were monitored every three days for illness clinical sign, body weight, survival rate, and blood parasitemia. Animal body weights were regularly measured during the course of the disease. PbA-infected and antioxidant-treated animals were assessed daily, and the time of death was promptly registered to determine the survival rate curve, as previously described by Oliveira et al. [24]. Parasitemia of individual mice was also measured by staining thin tail blood smears with 10% Giemsa solution (Sigma-Aldrich; diluted in PBS). Parasitemia (percentage of pRBCs) was evaluated by microscopic count and calculated as follows: [(number of pRBCs)/(total numbers of RBCs counted)] × 100.

### 2.4. Liver Histopathology

For histopathological analysis, at the 10^th^ day postinfection, PbA-infected and treated mice were first anesthetized with a solution of ketamine (100 mg/kg) and xylazine (10 mg/kg) and euthanatized by cervical dislocation as previously described by Okokon et al. [25]. The liver was aseptically collected by total hepatectomy, and samples were fixed in Bouin's solution (picric acid, 750 ml; formaldehyde, 250 ml; and acetic acid, 5 ml) for 48 hours. Then, they were progressively dehydrated with increased concentration of alcohol (70%, 80%, 90%, 95%, and 100%) for 40 minutes each. Subsequently, the samples were diaphanized in xylol twice for 30 minutes and paraffinized (58-60°C) for 60 minutes, forming paraffin blocks. Finally, the tissue blocks were cut with the aid of a microtome at a thickness of 5 *μ*m, followed by the assembly of histological slides. Then, deparaffinization was performed for 24 hours in a drying oven at 56°C, followed by hydration of the cuts through a sequence baths in xylol I, xylol II, absolute alcohol I, absolute alcohol II, and alcohol 95%, 90%, 80%, and 70% for three minutes each and distilled water for five minutes each. Slides were then stained with hematoxylin for 5 minutes and counterstained with eosin for one minute and, after successive washes, mounted on Permount (Fisher Scientific).

Liver sections were visualized under a light microscope (Nikon, Eclipse E800 Yokohama, Japan) and photographed at 200x magnification. Histological alterations in the tissue were semiquantitatively scored as described previously by Viriyavejakul et al. [26]. Briefly, double-blind analysis was performed in order to determinate the presence of steatosis, hyperplasic Kupffer cells, sinusoidal congestion, and hemozoin deposition. Each parameter was graded on a scale from 0 to 3: 0 = absent, 1 = mild, 2 = moderate, and 3 = severe. The total liver alteration score was expressed as the sum of the scores for each parameter with 12 being the maximum.

### 2.5. Biochemical Parameters of Liver Injury

The hepatocellular injury was assessed by evaluation of serum biomarkers of liver damage such as alanine aminotransferase (ALT), aspartate aminotransferase (AST), and total and direct bilirubin (BT and BD). Briefly, at the 10^th^ day postinfection, PbA-infected and treated animals were properly anesthetized and blood samples were collected by cardiac puncture. After centrifugation (3500 rpm for 10 minutes), the supernatant was removed and stored in a low-temperature freezer at –80°C until the biochemical test. Serum biochemical assays were conducted in accordance with the commercial kit instructions and determined in a spectrophotometer at a wavelength of 340 nm.

### 2.6. Statistical Analysis

Kaplan-Meier curves were generated for survival data, and significance was assessed by the Mantel-Cox logrank test. Parasitemia values were fitted by a linear function using the least square methods, and one-way ANOVA was conducted to determine the difference among the groups. Adjusted *p* values were calculated for multiplicity using the Bonferroni correction (*p* = 0.05/3). The level of plasma enzymes was expressed as the mean ± standard deviation (SD) and compared by two-way ANOVA, using Tukey-Kramer analysis as the posttest. All data were plotted and analyzed using the GraphPad Prism software (version 6.0), and *p* < 0.05 was considered statistically significant. All data are representative of at least three independent experiments.

## 3. Results

### 3.1. GSH and Vitamin C Treatments Modify the Survival Curve and Parasitemia Rate of PbA-Infected Mice

To describe the effect of GSH and vitamin C on the clinical progression of PbA-infected mice, animals were treated with 8 mg/kg/day of GSH and vitamin C, separately, for 7 consecutive days. As expected, during PbA infection, BALB/c mice developed characteristic clinical manifestations such as anemia, hepatosplenomegaly, and physic inactivity between the 17^th^ and 29^th^ days postinfection.

**Figure 1:**
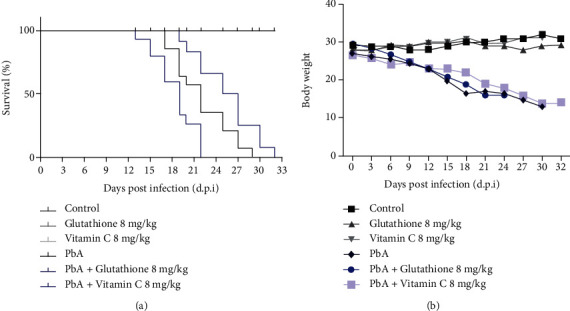
Glutathione and vitamin C treatments modify the survival curve of *Plasmodium berghei* ANKA-infected animals: (a) glutathione and vitamin C were administrated, intraperitoneally, at 8 mg/kg for 7 consecutive days, and survival curve analysis was carried out by using the Mantel-Cox logrank test (*p* ≤ 0.05); (b) body weight variation between groups during the infection (%). Results are representative of three independent experiments. Results shown are mean ± SD.

Our data showed that during PbA infection, nontreated animals were able to survive until 29 days postinfection; however, the treatment with 8 mg/kg GSH promoted a significant decrease in the survival of PbA-infected mice, with animals dying between days 17 and 19 postinfection (d.p.i). Furthermore, we also observed that 100% of PbA-infected animals treated with GSH died in the 23^rd^ d.p.i. while the PbA-infected group survived until the 29^th^ d.p.i. (Figure 1(a)).

On the other hand, vitamin C treatment promoted an improvement in the disease clinical outcomes and expanded the survival time of the PbA-infected group. Figure 1(a) demonstrates that 50% of animals treated with 8 mg/kg vitamin C died between the 25^th^ and 27^th^ d.p.i. and 100% only at the 34^th^ d.p.i. Further, GSH and vitamin C also had no effect on body weight average in PbA-infected mice and PbA-infected animals treated with antioxidants (Figure 1(b)).

**Figure 2:**
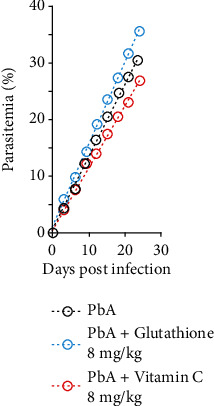
Course of *Plasmodium berghei* parasitemia in BALB-C mice treated with glutathione and vitamin C. Peripheral blood parasitemia was assessed by Giemsa staining. Parasitemia levels were measured as the number of parasitized red blood cells (pRBCs) in at least 1000 RBCs (two-way ANOVA; *p* = 0.05/3).

Our results have also shown that 8 mg/kg GSH promoted a fast and time-dependent increase in parasitemia values in PbA-infected animals (Figure 2). On the other hand, treatment with 8 mg/kg vitamin C has induced significant decrease in parasitemia values when compared with the nontreated PbA-infected group. This differential effect exerted by GSH and vitamin C can be better evidenced by mathematical analysis of parasitemia curve time evolution. As observed in Figure 2, PbA-infected animals which were treated with 8 mg/kg GSH or 8 mg/kg vitamin C present elevated and decreased values of the parasitemia saturation coefficient (1.46 and 1.09, respectively) when compared with the nontreated PbA-infected group (1.28).

### 3.2. GSH and Vitamin C Treatment, Respectively, Exacerbated and Attenuated Hepatic Histopathology Induced by PbA Infection in Mice

**Figure 3:**
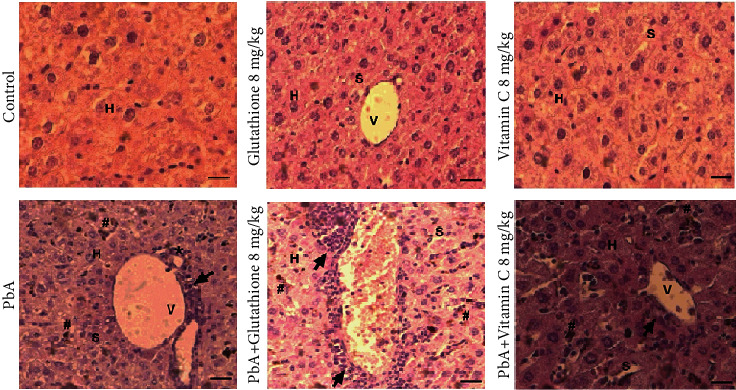
Histological alteration into the liver tissue of PbA-infected mice treated with glutathione and vitamin C. Representative light microphotographs from the liver of mice at day 10 postinfection and stained with H&E (×20 objective lens). Liver from uninfected mice (control group) with normal histological appearance and PbA-infected mice showing cellular infiltration (arrows) and vacuolation (asterisk) (bars, 20 *μ*m). H: hepatocytes; S: sinusoid; #: hemozoin.

To characterize liver histopathological alterations in PbA-infected animals treated with 8 mg/kg glutathione or 8 mg/kg vitamin C, we performed the hematoxylin-eosin staining (Figure 3). As shown in Figure 3, liver tissue sections from uninfected mice and uninfected mice treated with 8 mg/kg GSH or 8 mg/kg vitamin C had no morphological alterations, showing normal cellular architecture with distinct hepatic cells, sinusoidal spaces, and central veins. In PbA-infected animals, the sinusoidal area presented more enlargements, with deposition of hemozoin malaria pigment inside hyperplasic Kupffer cells and few spots with a discreet steatosis (Figure 3).

However, liver histopathological analysis of PbA-infected mice treated with 8 mg/kg GSH showed a more disarrangement of normal hepatic cells with hyperplasia and polymorphonuclear aggregation with increased numbers of inflammatory cells and vascular congestions (Figure 3). Increased cell infiltration including lymphocytes, mononuclear cells, and neutrophils was observed in the liver of PbA-infected mice treated with 8 mg/kg GSH on day 10 postinfection, leading to severe inflammation and tissue damage as evidenced in double-blind histological evaluation (Table 1). In contrast, animals treated with 8 mg/kg vitamin C presented less disarrangement of normal hepatic cells with few hyperplasia and polymorphonuclear aggregation, decreased numbers of inflammatory cells, and vascular congestions in comparison with nontreated PbA-infected mice. Vitamin C also decreased cell infiltration including lymphocytes, mononuclear cells, and neutrophils decreasing inflammatory response and liver damage in infected animals (Table 1).

### 3.3. Biochemical Measure of Liver Injuries in PbA-Infected Mice Treated with GSH and Vitamin C

**Figure 4:**
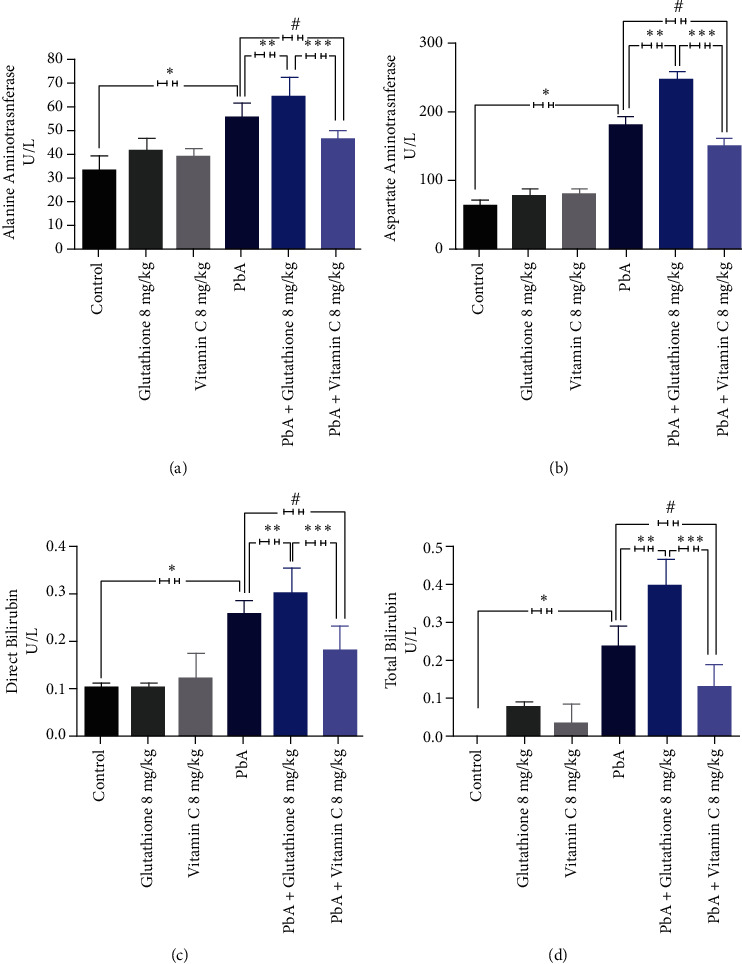
Liver enzyme activity in BALB-C mice infected with *Plasmodium berghei* ANKA and treated with glutathione and vitamin C. Alanine aminotransferase (a), aspartate aminotransferase (b), total bilirubin (c), and direct bilirubin (d) activities were determined 10 days postinfection in the serum of control uninfected animals, PbA-infected mice, and PbA-infected animals treated with glutathione and vitamin C. Data are presented as means ± SD with *p* ≤ 0.05.

To further assess functional changes, biochemical parameters of liver function were evaluated in the serum of uninfected control mice and PbA-infected mice treated with GSH or vitamin C. As shown in Figure 4, PbA-infected mice exhibited significant increase in serum AST, ALT, BD, and BT levels compared with the uninfected group (Figure 4). Moreover, PbA-infected mice treated with 8 mg/kg GSH showed elevated levels of serum AST, ALT, BD, and BT in comparison with both control and PbA-infected mice. However, our data have also shown that PbA-infected animals treated with vitamin C presented markedly reduced serum levels of AST, ALT, BD, and BT similar to those observed in the noninfected group (Figure 4), demonstrating that the treatment with vitamin C protects the liver from PbA infection injury.

## 4. Discussion

The current study demonstrated that treatment with antioxidants GSH and vitamin C induces a distinct effect on liver injuries caused by malaria infection. Results demonstrated that treatment with GSH accelerates the evolution of the disease in PbA-infected mice as well as the liver damage, while the treatment with vitamin C controls the disease outcome and mitigates the hepatotoxicity associated with the condition. It is widely described in literature that human malaria is characterized by intense hepatotoxicity and liver dysfunction, and although hepatic dysfunction in malaria is not fully understood, oxidative stress is often related to this condition [17]. As demonstrated in both human patients and animals models, liver damage during *Plasmodium* infection occurs as a result of free heme accumulation that triggers severe oxidative stress and stimulates proinflammatory response by tumor necrosis factor (TNF-*α*) release [27–29].

During malaria infection, liver dysfunction is often associated with elevated values of blood parasitemia [30–32], and data presented in our study show that mice infected with *Plasmodium berghei* ANKA demonstrated a time-dependent increase in parasitemia rates as well as intense liver damage as demonstrated by biochemical measurement (AST, ALT, and bilirubin levels). This is in agreement with the literature which describes a positive correlation between malaria hepatopathy and the activation of liver macrophages that phagocytize haemozoin or parasitized erythrocytes [12]. Our findings are also supported by previous studies demonstrating that the animal model represents a powerful and valuable tool to evaluate tissue and organ dysfunctions elicited by malaria [33, 34].

Data presented in our study have demonstrated that GSH treatment has favored infection and increased liver toxicity in PbA-infected mice. Although GSH represents an important antioxidant molecule in distinct tissue and organs, there are strong evidences demonstrating that species of the *Plasmodium* genus use GSH as a substrate for their reproduction in the vertebrate host [35–37]. Our results are in agreement with these reports since GSH treatment has induced a fast increase in parasitemia values; also, it has decreased animal survival in the PbA-infected group. Indeed, histological and biochemical evaluation in PbA-infected animals gives us strong evidences that GSH treatment exacerbates liver damage elicited by *Plasmodium* infection. Although more studies should be performed, these findings have relevant clinical implications for populations living in endemic malaria areas which largely use precursor of GSH synthesis such as acetylcysteine for the treatment of liver injuries.

While GSH treatment has favored PbA infection and liver impairment, our data also demonstrated that the treatment with vitamin C has a host beneficial effect in mice developing malaria. Anterior studies have already shown that vitamin C exerts a protective action in *Plasmodium*-infected animals [38–40]. Our study demonstrates that vitamin C treatment attenuates parasitemia evolution and prevents liver damage induced by malaria. Evidence of hepatic damage in the current study included events such as inflammation in the portal tract, hemozoin deposition, sinusoid congestion, and hyperplasia of Kupffer cells, and treatment with vitamin C proved to be effective in preventing these events. Our data are in accordance with previous reports which demonstrate the preventive effect of vitamin C on the hepatocytes in different models of liver injuries [41–43]. It is also well reported that alterations in the redox status of the liver is a mediating phenomenon of *Plasmodium* infection [44–46]. In this context, we hypothesized that vitamin C prevents liver damage in PbA-infected mice by avoiding oxidative stress induced by parasite infection.

Taken together, the data presented in the current study let us hypothesize that during *Plasmodium* infection, GSH and vitamin C are differently used in the host-parasite relation. In other words, while GSH could favor parasite reproduction and their life cycle, vitamin C acts preferentially in the host as a liver protective molecule.

## 5. Conclusions

In conclusion, this study demonstrated that, in BALB-C mice, the *Plasmodium berghei* ANKA strain induces a chronic infection and liver damage which could be differently modulated by the use of antioxidants. The treatment with GSH aggravated the disease outcome and liver injury, and, on the other hand, the treatment with vitamin C protects the liver from damage and the evolution of the disease.

## Figures and Tables

**Table 1:** Histopathological grading of liver lesions at 10 days post-*Plasmodium berghei* ANKA infection.

Histological changes	Histopathologic grading					
	Control	Glutathione (8 mg/kg)	Vitamin C (8 mg/kg)	PbA	PbA+glutathione (8 mg/kg)	PbA+vitamin C (8 mg/kg)
Fatty change	1	1	0	0	0	0
Kupffer cells	0	0	0	2	3	1
Sinusoid congestion	0	0	0	2	3	1
Hemozoin deposition	0	0	0	2	3	1
Portal tract inflammation	0	0	0	3	3	2
Bile duct proliferation	0	0	0	2	3	2
Total histological score	1/18	1/18	0/18	11/18	15/18	7/18

## Data Availability

The raw data supporting the conclusions of this manuscript will be made available by the authors, without undue reservation, to any qualified researcher.
